# Profile, knowledge, and work patterns of a cadre of maternal, newborn, and child health CHWs focusing on preventive and promotive services in Morogoro Region, Tanzania

**DOI:** 10.1186/s12960-015-0086-3

**Published:** 2015-12-24

**Authors:** Amnesty E. LeFevre, Rose Mpembeni, Dereck Chitama, Asha S. George, Diwakar Mohan, David P Urassa, Shivam Gupta, Isabelle Feldhaus, Audrey Pereira, Charles Kilewo, Joy J Chebet, Chelsea M Cooper, Giulia Besana, Harriet Lutale, Dunstan Bishanga, Emmanuel Mtete, Helen Semu, Abdullah H Baqui, Japhet Killewo, Peter J Winch

**Affiliations:** Department of International Health, Johns Hopkins Bloomberg School of Public Health, Baltimore, MD USA; School of Public Health and Social Sciences, Muhimbili University of Health and Allied Sciences, P.O. Box 65015, Dar es Salaam, Tanzania; Jhpiego, 1615 Thames Street, Baltimore, MD 21231-3492 USA; Ministry of Health and Social Welfare, Dar es Salaam, Tanzania; International Center for Maternal and Newborn Health, Department of International Health, Johns Hopkins Bloomberg School of Public Health, Baltimore, MD USA

**Keywords:** Community health workers, Maternal newborn child health, Tanzania

## Abstract

**Background:**

Despite impressive decreases in under-five mortality, progress in reducing maternal and neonatal mortality in Tanzania has been slow. We present an evaluation of a cadre of maternal, newborn, and child health community health worker (MNCH CHW) focused on preventive and promotive services during the antenatal and postpartum periods in Morogoro Region, Tanzania. Study findings review the effect of several critical design elements on knowledge, time allocation, service delivery, satisfaction, and motivation.

**Methods:**

A quantitative survey on service delivery and knowledge was administered to 228 (of 238 trained) MNCH CHWs. Results are compared against surveys administered to (1) providers in nine health centers (*n* = 88) and (2) CHWs (*n* = 53) identified in the same districts prior to the program’s start. Service delivery outputs were measured by register data and through a time motion study conducted among a sub-sample of 33 randomly selected MNCH CHWs.

**Results:**

Ninety-seven percent of MNCH CHWs (*n* = 228) were interviewed: 55% male, 58% married, and 52% with secondary school education or higher. MNCH CHWs when compared to earlier CHWs were more likely to be unmarried, younger, and more educated. Mean MNCH CHW knowledge scores were <50% for 8 of 10 MNCH domains assessed and comparable to those observed for health center providers but lower than those for earlier CHWs. MNCH CHWs reported covering a mean of 186 households and were observed to provide MNCH services for 5 h weekly. Attendance of monthly facility-based supervision meetings was nearly universal and focused largely on registers, yet data quality assessments highlighted inconsistencies. Despite program plans to provide financial incentives and bicycles for transport, only 56% of CHWs had received financial incentives and none received bicycles.

**Conclusions:**

Initial rollout of MNCH CHWs yields important insights into addressing program challenges. The social profile of CHWs was not significantly associated with knowledge or service delivery, suggesting a broader range of community members could be recruited as CHWs. MNCH CHW time spent on service delivery was limited but comparable to the financial incentives received. Service delivery registers need to be simplified to reduce inconsistencies and yet expanded to include indicators on the timing of antenatal and postpartum visits.

**Electronic supplementary material:**

The online version of this article (doi:10.1186/s12960-015-0086-3) contains supplementary material, which is available to authorized users.

## Background

Child mortality rates in Tanzania have declined by nearly 70% over the last 25 years and in 2014 nearly reached the Millennium Development Goal (MDG) 4 target of 55 per 1000 live births [1,2]. At the same time, progress in reducing neonatal and maternal mortality has been slower. Despite a 35% decline in neonatal mortality from 1991 to 2014, deaths within the first 28 days of life comprise half of child deaths [1,2]. Among mothers, mortality has declined by 55% since 1991, and yet, with 410 maternal deaths per 100 000 live births reported in 2014, progress towards MDG 5 has been insufficient to reach the target of 230 per 100 000 live births [3].

Key contributors to the slow progress in reducing neonatal and maternal mortality have been (1) stagnant levels of facility deliveries, (2) poor quality of care, (3) lack of contact of health services with children during their first 28 days of life, and (4) breaks in the continuity of care from preconception through antenatal, intra-partum, and postpartum periods. Critical shortages of health workers underpin these contributing factors, impeding efforts to improve timely and continuous access to high-quality health services in Tanzania and many other low-resource settings. When compared to the WHO-recommended health workforce density of 25 health professionals (including physicians, nurses, midwives) per 10 000 people, Tanzania lags behind with only 4 health professionals for every 10 000 citizens [2]. The difficulties in addressing the challenges underpinning human resources for health in Tanzania, including inadequate training and recruitment, uneven workforce distributions, and retention, coupled with the desire to extend the reach of health services, have led to a proliferation of community health worker (CHW) programs [4].

The use of CHWs in Tanzania began in the 1960s alongside President Nyerere’s implementation of *ujamaa* (collectivized) villages, which in part aimed to make social services more accessible to rural populations [5]. Early programs sought to train medical auxiliaries and village medical helpers (VMH), selected and supported by communities [5]. However, these programs were limited in their scale and effectiveness. By 1978, only 2000 VMHs had been trained, and findings from an evaluation of the health sector found limited evidence of community involvement in planning and program monitoring [5]. In 1983, the Ministry of Health and Social Welfare (MoHSW) released CHW training guidelines with the intent of piloting small-scale programs in 10 districts and ultimately establishing two VMHs (one male, one female) and a health post in each village [5]. These plans were not realized, and instead, three decades of fragmented CHW programs ensued, each varying in scale, training content and duration, and engagement with the health system and community.

Since the 1980s, CHW programs in Tanzania have concentrated heavily on narrow mandates determined by funders, often focusing on only one health problem, most commonly HIV/AIDS [6,7]. While some consistency has existed in terms of CHW requirements, including literacy and community selection, the geographical coverage of programs, training intensity, and curriculum used has varied widely. To date, a system for integrating cadres of community-based providers into the formal health sector in Tanzania has yet to be developed. More recently, the government has explicitly mentioned CHWs as an integral aspect of its health improvement strategy in the Primary Health Services Development Program (PHSDP) of 2007 and the National Road Map Strategic Plan to Accelerate Reduction of Maternal, Newborn and Child Deaths in Tanzania [8]. In 2013, a national CHW task force was created by the MoHSW to achieve consensus on the development of a national cadre of CHWs and establish a foundation for the training of CHWs.

In 2012, the MoHSW approved national guidelines for training maternal, newborn, and child health (MNCH) CHWs as part of an interim solution until a single national cadre of CHWs could be established. These volunteer MNCH CHWs are selected by the community, trained for 21 days, and supervised by facility providers to deliver a range of preventive and promotive services during the antenatal and postpartum periods through home visits and community meetings. This socially orientated CHW is in contrast to the more medically orientated integrated community case management (iCCM) CHW models being established in a number of other countries in the region [9].

These CHW model variations require different provider competencies. For iCCM CHWs to be effective, CHWs need to be able to correctly identify, assess, and treat sick children based on national guidelines, and systems need to be in place to ensure adequate clinical supervision and medical supplies. In contrast, for a preventive-promotive MNCH CHW to be effective, it is necessary to master a large amount of information, understand which messages are appropriate for visits at different points in the continuum of care, and reach a high proportion of women and children at each point in the continuum. For more comprehensive CHW models, which draw upon a range of both medically and socially orientated tasks, CHWs must achieve competency in all of these tasks.

This paper profiles a recently established cadre of socially oriented MNCH CHWs that provides health preventive and promotive MNCH services in Morogoro Region, Tanzania. We review several critical design elements including CHW profile and MNCH knowledge, CHW to population coverage ratio, and program monitoring and supervision and assess their implications on time allocation, service delivery, satisfaction and motivation, and incentive preferences.

## Methods

### Study setting

Two hundred kilometers west of Dar es Salaam, Morogoro Region is home to over 2.2 million people dispersed over 70 000 km^2^, making it the sixth most populous and second largest of the country’s 25 mainland regions [10]. Seventy-three percent of Morogoro Region is rural with regional averages for education, poverty, and care seeking similar to national averages [10]. Over half of the population (51%) falls within the middle to upper middle wealth quintiles, as compared to 42% on a national level [11]. In the health sector, trends in care seeking for critical MNCH services mirror national trends for postnatal care (35%) and are slightly higher than national averages for most other indicators, including antenatal care (ANC) utilization (98% versus 96%), facility deliveries (58% versus 50%), and skilled birth attendance (61% versus 51%) [11].

### MNCH CHW program and evaluation

Implemented by the MoHSW with support from Jhpiego and established through the USAID-funded Mothers and Infants, Safe, Healthy and Alive (MAISHA) program, the Integrated Community to Facility MNCH Program aims to improve access to and quality of maternal, newborn, and reproductive health services. Integrated MNCH CHW Program training activities began in 2010 with a 6-day training of health center providers (mean of 2–4 per facility) according to facility-based guidelines (Table 1). In 2012, in districts where facility-based training occurred, 2 health centers and 10 dispensaries (5 dispensaries per health center) were selected as sites for the MNCH CHW program. For each health center or dispensary selected, two villages were identified and asked to nominate one male and one female resident with ideally secondary school education to serve as MNCH CHWs (Additional file 1: Figure S1). Selected CHWs received training for 21 days on behavior change, interpersonal communication and counseling, care during pregnancy, maternal postpartum care, newborn and child care, infant and young child feeding, community-based family planning, prevention of mother to child transmission, community involvement and participation, the integrated management cascade and supportive supervision, and monitoring and evaluation. Following training, CHWs were deployed to their home communities to conduct surveillance for pregnancy and delivery and provide counseling during three pregnancy and six postpartum home visits. Counseling was intended to elicit adoption of optimal health practices and promote the use of MNCH services among pregnant, postpartum women and their support networks (including partners and other members of the community). MNCH CHWs were supervised by trained facility-based dispensary and health center providers (enrolled nurses and/or clinical officers) through monthly supportive supervision visits and by MoHSW (regional and district) and Jhpiego staff on a quarterly basis. Supervision visits focused on a review of registers and reporting forms for data quality, activity planning, and a review of achievements and planning. Additional details on the content and effect of supervisory activities are presented elsewhere [12].

**Table 1:** **MNCH CHW program implementation strategy**

*Program design*	
• Needs assessment	
• Results dissemination: dissemination of needs assessment findings to key regional/district staff	
*Program rollout*	
• Regional advocacy meetings: 1–2-day meetings to explain the program and cover expectations among regional/district staff	
• Community-based advocacy to inform village leadership on what is needed and criteria, program objectives, and support required (expectations)	
*Capacity building*	
• Training of trainers, final pretest, and package review: 21-day training for approximately 25 people, including 5 trainers, 5 district representative, MOHSW, and Jhpiego personnel	
• Supervisor training: 14 days per batch, including 1–2 providers per facility, MOHSW, and Jhpiego staff	
• CHW training: 2–4 CHWs per village, 21 days of training according to National MNCH CHW guidelines	
*Implementation monitoring and support*	
• Community HMIS system established	
• Quarterly facility supervision: 1 day per health center/dispensary, supervision carried out by Jhpiego staff (1–2), regional and district MOHSW to support service delivery at health centers	
• Community supervision: 1 day per health center/dispensary overseeing 2 villages of CHWs; supervision carried out	
o Quarterly by regional and district MOHSW and Jhpiego staff (1–2)	
o Monthly by routine facility-based supervisors	

### Study design and sampling

Table 2 summarizes data sources. The evaluation of MNCH CHWs sought to determine their profile and MNCH knowledge, CHW to population coverage ratio, program monitoring and supervision, incentives, satisfaction and motivation, and service delivery.

**Table 2:** **Data sources for assessing outputs of MNCH CHW program activities in five districts of Morogoro, Tanzania**

**Outputs**	**Measurement methods**	**Date of collection**	**Sampling**	**Final sample**
Knowledge	Community health worker survey and census	September to October 2011	100% of all identified CHWs in 5 districts of Morogoro	*n* = 53 CHWs from 10 villages
	Structured interviews with health center reproductive child health (RCH) providers	September–October 2012	Interviews with health center RCH providers (*n* = 88) available on day of visit	9 health centers in 5 districts of Morogoro
				88 RCH providers
	MNCH CHW survey	September to October 2013	238 MNCH CHWs trained by end of July 31, 2014	97% of MNCH CHWs (*n* = 228) trained by July 31, 2014, in 79 villages
Supervision				
Establishment of HMIS tracking systems	MNCH CHW HMIS service delivery data	September to October 2013	Review and extraction of HMIS data from 238 MNCH CHWs for the previous 5 months	Summary register data for May to July 2013 from 228 (97%) MNCH CHWs
Reported home visits				
Observed service delivery	Direct observations of MNCH CHWs	December 2013 to January 2014	10% of 228 MNCH CHWs trained by July 31, 2013, randomly selected	*N* = 33 CHWs observed for 6 consecutive days from Wednesday to Monday

To determine the MNCH CHW profile, knowledge, supervision, and service delivery outputs, a quantitative survey drawing from the MoHSW MNCH national guidelines on the content of training provided was administered to 228 (of the 238) MNCH CHWs following their recruitment, training, and deployment (Table 3). MNCH CHWs trained at least 3 months (from December 2012 to July 2013) prior to the start of the survey in October 2013 were eligible for inclusion. If participants were unavailable during researchers’ first visit to a village, a return visit for the interview was arranged at a later date during the period of data collection. Participants were not included if they did not consent to the interview, dropped out of the program, were traveling with an unknown return date, sick/hospitalized, or deceased at the time of data collection. The survey administered to consenting individuals included sections on CHW socio-demographics, service delivery, supervision, incentives, satisfaction, motivation, and MNCH knowledge. The latter included 38 questions with 191 possible responses (unprompted) across the following domains: pregnancy (3 questions), postpartum (3 questions), newborn care (3 questions), child health (7 questions), nutrition (4 questions), HIV transmission (3 questions), malaria (1 question), infection prevention (3 questions), injury prevention 1 (question), and family planning (10 questions), all of which aligned with the CHW curriculum. The average number of correct responses was used to generate a composite score for each domain and an overall average derived from across the averages calculated for each of the 10 domains (mean of means).

**Table 3:** **MNCH CHW profile and characteristics**

	***N*** ** = 228**	**Percent**
Date of training		
Dec 2012–Jan 2013	46	20
April–May 2013	86	38
July 2013	94	42
Gender		
Male	125	55
Female	103	45
Age (mean/median/range)	(33/32/19–61)	
<25 years	68	30
25–35	75	33
>35	85	37
Marital status		
Married	133	58
Not married	73	32
Other: cohabiting, widowed, divorced	22	10
Education (median years)		
Primary started	4	2
Primary completed	104	46
Form 4 or higher	114	52
Ability to read		
Ability to read some	1	0.4
Ability to read all sentence	227	99.6
Languages spoken fluently		
Swahili	228	100
Local language	192	84
English	34	15
Number of dependents (mean/median/range)	(3.28/3/0–12)	
Income-generating activities (multiple options possible)		
Agriculture		
Crops	213	93
Livestock	37	16
External employment		
Government	0	0
Private sector	2	1
Self employed	27	12
Not working outside the home	15	7
Household income per monthly all sources (mean/median/range)	($48/$31/$0–$305)	

MNCH CHW knowledge results were compared against knowledge surveys administered to two populations of providers operating in the same geographic area: (1) health center reproductive child health (RCH) providers (*n* = 88) and (2) CHWs identified in the same districts prior to the program’s start at the community level (*n* = 53). These comparisons were intended to spur discourse on MNCH CHW eligibility criteria and provide broader insights into MNCH CHW competency and service delivery. RCH providers in nine health centers (*n* = 88) were interviewed during a facility assessment survey conducted in 2012. In 2011, prior to the rollout of MNCH CHWs, a CHW census was carried out to determine the number of providers and assess knowledge and service delivery of individuals who self reported and/or were said to be CHWs (*n* = 53) by key stakeholders including village leaders and facility-based providers. Once identified, research assistants administered a quantitative survey exploring personal characteristics, working conditions, incentives, knowledge, motivation, and job satisfaction.

Service delivery outputs were measured by extracting data from the Health and Management Information System (HMIS) registers of interviewed MNCH CHWs for the 5 months preceding the survey (May to September 2013) and through direct observations. For the latter, a time motion study was conducted from December 2013 to January 2014 among a sub-sample of ~15% (*n* = 33) of MNCH CHWs randomly selected from among those interviewed for the quantitative survey. Observations sought to improve understanding of the frequency and content of MNCH service provision, including use of job aids^a^, as well as the broader context within which services are provided. The time motion study was constrained to the CHW’s village of residence and spanned for a period of up to six consecutive days beginning on a Wednesday and ending on a Monday in most instances. During the period of observation, a team of independent research assistants observed and continuously timed all activities carried out between the hours of 8 am and 5 pm. Activities performed outside of the observation window (from 5 pm to 8 am) were self-quantified at the start of each new day and recorded as “reported time allocation.” Findings are presented only on observed time allocation.

To assess the quality of HMIS registers, among the MNCH CHWs observed during the time motion study (*n* = 33), we compared MNCH monthly summary sheets for 3 months with the maternal and child health (MCH) registers for the same 3 months for each of the 33 CHWs. Summary sheets form the basis of reported service delivery statistics and are submitted by individual MNCH CHWs to supervisors monthly and ultimately aggregated across all MNCH CHWs. The MNCH CHW summary sheets were assessed for discrepancies (over or under reporting) with the MCH registers in the number of the following visits: new pregnant women, returning pregnant women, neonates, children 1–12 months old, children 12–59 months old, and total home visits. For each type of visit, we calculated the number of CHWs with discrepancies and the magnitude of these discrepancies. We also assessed for patterns of discrepancies by CHW gender, education, and date of training.

### Data analyses

Quantitative data were double entered and cleaned using Epi Info software, with statistical analyses performed using Stata 12.0. Summary composite scores for knowledge were calculated by taking the average number of correct responses for each domain and then an overall average across the averages calculated for each of the 10 domains (mean of means). Ordered logistic regression models were used to explore associations between MNCH CHW characteristics (gender, age, education, assets, date of training) and composite knowledge scores overall and across domains. An asset index was constructed from CHW household assets and characteristics, using principal components analysis. Time motion data were analyzed using basic frequencies and cross tabulations.

### Ethical approval

The study received ethical approval from the Muhimbili University of Health and Allied Sciences and Johns Hopkins School of Public Health Institutional Review Boards. Preliminary findings were shared with key decision makers in Tanzania from the MoHSW and Jhpiego for their feedback and review prior to publications being drafted.

## Results

### CHW profile

Ninety-seven percent of the CHWs (*n* = 228) reported as trained between December 2012 and July 2013 were identified and successfully interviewed based on personnel lists provided by the Integrated MNCH CHW Program. Fifty-five percent of MNCH CHWs were male, all were Swahili speakers (100%), 58% were married, and 52% had secondary school education or higher (Table 2). The mean monthly household income from all sources was equivalent to US$ 47.61 (range: $0–$305.25), and nearly all reported generating income through agriculture (93% grew crops, 16% had livestock).

When compared against CHWs interviewed in 2011 (*n* = 53), prior to the start of MNCH CHW program, the ratio of male to female CHWs was similar over time. However, differences in age, education, and marital status were observed. In 2011, CHWs were more likely to be married (75% in 2011 versus 58% in 2013), were older (mean age of 41 in 2011 as compared to 32 in 2013), and were less educated (17% had secondary school education or higher in 2011 as compared to 52% in 2013).

### Knowledge

On average, CHWs were able to recall correctly and unprompted 47% of responses to 38 questions across 10 domains of pregnancy care, postpartum care for mothers and newborns, child health, nutrition, HIV, malaria, family planning, infection, and injury prevention. Among the 10 domains assessed, recall of family planning messages was highest (73%), while postpartum care (40%), HIV transmission (37%), and nutrition (35%) were the lowest (Additional file 1: Figure S2).

Ordered logistic regression models sought to explore the association between personal and program characteristics on composite scores for overall knowledge and specific domains of pregnancy, postpartum, newborn care, and child health (Additional file 1: Tables S1 and S2). Among domains, CHWs who were trained more recently had significantly higher odds of recalling critical indicators for pregnancy, family planning, infection, injury prevention, and nutrition as compared to individuals trained in December/January 2013.

**Figure 1:**
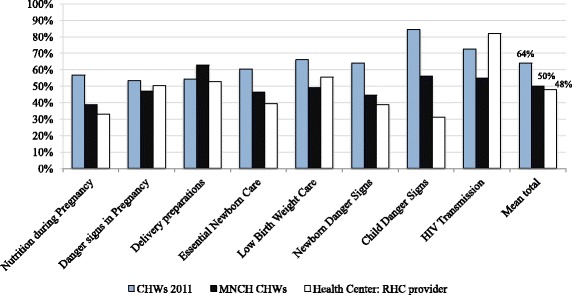
**Comparison of MNCH knowledge: (1) self-identified CHWs from 2011, (2) MNCH CHWs, and (3) reproductive child health providers in health centers.**

When compared against health center RCH providers and CHWs interviewed in the same districts from 2011, descriptive trends suggest that mean knowledge scores were similar for health center RCH providers (48%) and MNCH CHWs (50%) but lower than those for CHWs interviewed in 2011 (64%) (Figure 1).

### CHW to population ratio

CHWs reported having a mean of 186 (median of 120; range 3 to 1702) households per catchment area to cover, corresponding to approximately 1 CHW per 967 people (median 1 per 624 population). Assuming a birth rate of 30 per 1000, this corresponds to an estimated 29^b^ pregnancies and 27 newborns annually. With three visits during pregnancy, four during the first 28 days of life, and two within 1–59 months, CHWs would need to make an estimated 39 visits monthly (7 pregnancy visits, 9 postnatal, and 23 among children 1–59 months). Assuming a 20-day work month, CHWs would need to conduct almost two household visits per day. In the event of an 8-day work month, CHWs would be required to conduct nearly five household visits per day.

### Monitoring CHW service delivery

Systems for tracking MNCH CHW service delivery were established through the introduction of MNCH CHW HMIS registers: (1) maternal and child health (MCH) and (2) referral register. Key summary indicators for the former are outlined in Table 4.

**Table 4:** **CHW self-reported activities from MNCH CHW survey**

	***N*** ** = 228**
Duration working as MNCH CHW in months (mean/median/range)	1.82/2/1–5
Workload	
Households served by MNCH CHW (mean/median/range)	186/120/3–1702
Days per week providing services	2.9/3/0–7
Hours per day (mean/median/range)	4.78/5/1–6
Links with other CHWs and other programs	
Other CHWs working in the same area (mean/median/range)	3.7/4/1–4
Other MNCH CHWs working in the same area (mean/median/range)	1.03/0/0–10
Work with other CHW programs	18%
Work in health facilities (multiple responses possible)	71%
Recording keeping	54%
Weighing children	51%
Referral	33%
Home follow-up	24%
Deworming support	22%
Vaccination support	20%
Vitamin A support	20%
Distance from home to facility in km (mean/median/range)	4.8/3/0–50
Mode of transportation	
Foot	70%
Bicycle	22%
Motorbike	8%

Seventy-eight percent of MNCH CHWs interviewed in the quantitative survey (*n* = 228) was currently recording information into the MCH register, and 98% was recording information in the referral register. For the MNCH CHWs observed during the time motion study (*n* = 33), summary sheets for a 3-month period generated by trained research assistants were compared against those recorded by MNCH CHWs. The quality of data was found to be variable across the eight indicators (type of visits) assessed (Table 5). Comparing monthly summaries with the mother and child register found that 8 (24%) CHWs had a discrepant number of visits to new pregnant women, while 18 (55%) CHWs had a discrepant number of households visited. The CHW’s gender, education level, and date of training were not significantly associated with these discrepancies for any of the eight indicators (visits). No systematic pattern of over or under reporting for these discrepancies was observed when assessed by source of data (summary sheet versus the monthly register). The magnitude of these discrepancies ranged from a low of 7 for neonatal visits to 92 for number of children from 1 to 5 years visited in any given month.

**Table 5:** **Comparison of monthly summary register with mother and child register**

**Indicator (** ***N*** ** = 33)**	**Number of CHWs with discrepant entries (percent)**	**Average number of months with discrepant entries (range 0–3 months)**	**Magnitude of discrepancy (Range over 3 months)**
Number of new pregnant women visited	8 (24.2%)	1.5	1–9
Number of returning pregnant women visited	14 (42.4%)	1.4	1–7
Number of neonatal visits	15 (45.4%)	1.5	1–6
Number of children from 1 month up to 1 year visited this month	16 (48.4%)	1.5	1–7
Number of children from 1 year to 5 years visited this month	16 (48.4%)	2.3	1–92
Number of households visited	18 (54.5%)	1.8	1–20

**Figure 2:**
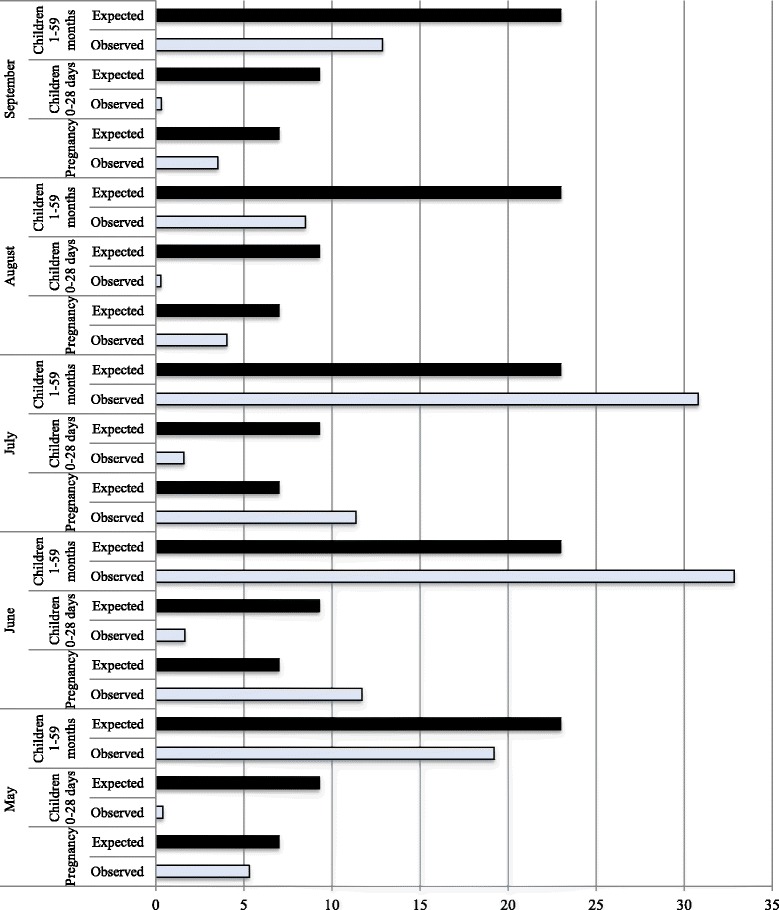
**Expected versus observed home visits among pregnant women, postnatal, and children 1–59 months.**

Despite challenges in MNCH CHW reporting of service delivery, a review of HMIS records provides insights into service delivery. On a monthly basis, CHWs reported providing MNCH services to a mean of 15–21 households (median of 12–14), which is approximately between 2–3 households per day during a median 2 days of work per week (Table 6). Of the MNCH home visits carried out, HMIS records suggest that nearly 44% were made to children 12–59 months, 29% to women during pregnancy/postpartum, 20% to infants 1–11 months, and 8% to newborns (Table 6). Figure 2 juxtaposes expected and observed numbers of home visits by month. Deficits between expected and observed home visits were most pronounced amongst newborns (0-28 days). Among pregnant women and children 1-59 months, observed home visits exceeded expected numbers only for the months of June and July.

**Table 6:** **MNCH CHW monthly service delivery from May-September 2013**

	**May**	**June**	**July**	**August**	**September**
	***n*** ** = 121**	***n*** ** = 123**	***n*** ** = 127**	***n*** ** = 200**	***n*** ** = 212**
	**Mean/median (range)**	**Mean/median (range)**	**Mean/median (range)**	**Mean/median (range)**	**Mean/median (range)**
Women clients					
Number of women visited	7/7 (0–44)	7/5 (0–30)	7/6 (0–22)	5/4 (0–21)	5/4 (0–22)
New pregnant women visited	4/3 (0–19)	2/2 (0–13)	2/2 (0–9)	3/2 (0–18)	2/2 (0–16)
Returning pregnant women visited	2/1 (0–9)	2/2 (0–12)	2/2 (0–14)	1/1 (0–11)	1/1 (0–11)
Number of women visited after delivery	2/2 (0–34)	2/1 (0–11)	2/1 (0–10)	1/1 (0–10)	2/1 (0–10)
Newborn and child clients					
Number of newborns/infants/under 5s visited	22/14 (0–162)	21/15 (0–202)	19/13 (0–196)	16/11 (0–175)	15/11 (0–79)
Number of neonates (under 1 month) visited	2/2 (0–14)	2/2 (0–30)	2/1 (0–10)	2/1 (0–9)	2/1/ (0–12)
Number of children from 1 month to 1 year visited	5/4 (0–33)	5/4 (0–28)	5/3 (0–31)	4/3 (0–30)	4/3 (0–21)
Number of children from 1 year to 5 years visited	14/7 (0–136)	13/9 (0–168)	12/7 (0–166)	10/6 (0–148)	9/6 (0–60)
Referrals					
Number of referrals	1/0 (0–13)	1/0 (0–18)	0.8/0 (0–6)	0.7/0 (0–10)	1/0 (0–8)
Number of women referred to a health facility	0.5/0 (0–7)	0.5/0 (0–6)	0.4/0 (0–6)	0.4/0 (0–6)	0.5/0 (0–6)
Number of neonates referred to a health facility	0.2/0 (0–3)	0.2/0 (0–5)	0.1/0 (0–2)	0.1/0 (0–6)	0.2/0 (0–4)
Number of children from 1 year to 5 years referred to a health facility	0.3/0 (0–5)	0.4/0 (0–9)	0.2/0 (0–6)	0.2/0 (0–6)	0.3/0 (0–5)
Households					
Number of households visited	21/14 (0–207)	21/14 (0–188)	18/14 (0–188)	17/11 (0–190)	15/12 (0–99)
Health education meetings					
Number of health education meetings conducted	0.7/0 (0–9)	0.4/0 (0–3)	0.5/0 (0–4)	0.5/0 (0–6)	0.5/0 (0–3)
Number of people attending meetings	36/0 (0–415)	20/0 (0–287)	17/0 (0–140)	29/0 (0–600)	20/0 (0–178)

CHWs reported having at least one other MNCH CHW and an average of 3.7 other CHWs working in the same area. A significant association between mean households served and mean monthly home visits reported was not observed (Pearson’s correlation coefficient of 0.0128, *P* < 0.8). However, trends in the mean number of households visited per month per CHW were observed to decline slightly over time with the concurrent growth in the number of MNCH CHWs providing services.

### Supervision of CHW performance

All interviewed CHWs (*n* = 228) reported attending monthly supervision meetings held in health centers or dispensaries continuously since receipt of training. High rates of attendance may in part be attributed to financial incentives disbursed during these monthly meetings (25,000 TSH; US$ 15.00) and quarterly supervision visits (10,000 TSH (US$ 6.00)) which total 50% of the MNCH CHWs’ median monthly household income (50,000 TSH).

Facility-based providers also were reported to visit CHWs in the village a mean of once in 2 months. Planned quarterly supervision from Jhpiego/MoHSW regional and district providers occurred with less frequency. Among CHWs (*n* = 46) trained in December 2012/January 2013, a mean of 1.5 of the three scheduled quarterly visits had taken place. Nearly all CHWs trained (*n* = 86) in April/May 2013 had received their scheduled quarterly supervision visit (CHWs reported a mean of 0.76 quarterly visits).

CHWs were asked to provide details on the content of supervision; multiple responses were allowed. During both monthly and quarterly supervision visits, focus on checking the content of HMIS registers was mentioned with the greatest frequency (>80%). Knowledge assessments, feedback on work performance, work planning, and/or additional training were mentioned with less frequency (<50%).

### MNCH service delivery

Thirty-three MNCH CHWs were observed: 29 for a total of 6 days and the remaining for less than 5 days. MNCH CHWs were directly observed to spend 6.75 (15%) out of 43 h on health service delivery, 11% (5 h) on MNCH services, and 4% (1.75 h) on other health work. Given the financial incentives paid by the MNCH CHW program, time spent on the MNCH CHW program corresponds to 1,750 TSH or US$ 1.06 per hour and compares favorably to the estimated hourly wage of 1,805 TSH or US$ 1.09 earned through time spent on alternative income-generating activities.

Of the 5 h CHWs spent on MNCH activities, 70% was indirectly related to client care (supervision meetings 40%, travel 23%, and registers 7%) and 30% on home visit consultations. CHWs were observed to provide home visits to a mean of two clients per week with each visit ranging from 75 min for a pregnancy visit, 86 min for a postpartum visit, and 19 min for a follow-up visit made 5 months after the immediate postpartum period.

### Job aids

During 37 observed pregnancy visits, 26 different job aides were used, 3 with ~80% frequency: individual birth preparedness (IBP), pregnancy danger signs, and maternal nutrition. Two job aids recommended for use during all pregnancy visits—pregnancy danger signs and prevention of mother to child transmission—were missed in 19% and 38% of home visits, respectively. Thirty-three percent of job aids observed were not those recommended for pregnancy visits but instead cited as optional content. During nine observed postpartum visits, 14 different job aides were observed, 6 with nearly 80% frequency: malaria prevention, lactational amenorrhea method, nutrition, child danger signs, accident prevention, and infection prevention. The single job aid on breastfeeding recommended for use in all postpartum home visits was missed in 23% of visits.

### MNCH CHW motivation, satisfaction, and incentives

Almost all (99%) CHWs interviewed reported being happy to work as CHWs and over 90% felt their work to be valued by both the health facility workers and the community. High levels of satisfaction were reported for the availability of job aids (90%) and registers (91%), level and quality of training received (90%), and quality of their own work (88%) [13]. Almost all (93%) were unsatisfied with the availability of transport used for care provision and for travel to the health facility^a^, and 80% of CHWs were dissatisfied with financial incentives provided [13].

MNCH CHWs were intended to receive 25,000 TSH (US$ 15.00) for attending monthly meetings and 10,000 TSH (US$ 6.00) for attendance of quarterly visits from Jhpiego and regional and district MoHSW staff. When provided, this amount corresponds to an estimated 50% of the median monthly household income of 50,000 TSH (US$ 28.78) reported by CHWs. When asked about incentives received, slightly more than half (56%) of CHWs had received financial incentives since their training, but none had received the bicycles promised to facilitate transport. When asked their preference, non-monetary incentives are relatively more important than monetary incentives for CHWs. Specifically, community recognition and respect were most preferred over other incentives. However, CHW remuneration and retention remain a concern, as 60% of CHWs feel overburdened due to other competing household and professional responsibilities to the extent that 14% indicate that they contemplated quitting. Younger CHWs were more likely to feel overburdened and less valued by community members.

## Discussion

Building upon five decades of CHW programs in Tanzania, the Integrated MNCH CHW Program sought to establish a volunteer cadre of MNCH CHWs that provide a range of socially oriented, preventive, and promotive services including village mapping, pregnancy surveillance, counseling through home visits, and health promotion meetings. This model is in contrast to other current CHW initiatives like the iCCM model, which expands on preventive and promotive activities to include curative services, requiring clinical oversight, training, and support inclusive of more sophisticated supply chain mechanisms. We examine how time allocation, service delivery, satisfaction, and motivation are effected by the following critical design elements (1) CHW profile and MNCH knowledge, (2) CHW to population ratio, (3) program monitoring and supervision, and (4) incentives.

CHW characteristics, including age, gender, education, and martial status, may influence performance [14,15]. At inception, the Integrated MNCH CHW program sought to train an equal proportion of male/female CHWs, with secondary school or higher education, who resided in and were selected by the communities where they would eventually work. In practice, MNCH CHWs were nearly evenly split in gender (55% male, 45% female), under 35 years of age (63%), nearly one third was unmarried, and only half met the MoHSW requirement of Form 4, secondary education or higher. Elsewhere globally, the sex of CHWs has been show to influence the reported frequency of counseling [16] and uptake of services, particularly for reproductive health [17] and child nutrition [18], as well as record keeping [16]. Education has been listed as an influencing factor in five prior CHW studies, and while higher education may lead to better performance, it may also correspond to higher rates of attrition [14]. Studies exploring the influence of age on performance have found evidence of poorer performance among younger and older CHWs [14]. In Kenya, the optimal CHW age range was 30–40 years [16]. While we explore the implications of these social characteristics elsewhere (Intersectionality implications of scaling up MNCH CHVs in Tanzania: examining how gender, age and educational determinants combine to influence CHV experience, to be submitted.), further analyses found no significant differences in the mean number of households visited monthly (service delivery) and in the mean composite scores for overall knowledge by CHW education, gender, or age, although qualitative data indicated that CHW education, gender, and age did influence CHW communication and visits with community members [19].

Overall mean knowledge scores for MNCH CHWs were observed to be poor at less than 50% for 8 of 10 MNCH domains assessed. However, set standards for interpreting knowledge scores for CHWs are not available within the literature to enable comparison between studies. Job aids may temper the effect of these gaps in CHW knowledge during counseling, and supportive supervision and fresher training help to overcome them. However, additional efforts are needed to prioritize key messages based on evidence and to assess whether changes in content or duration of training might lead to improvements in knowledge, ultimately translating to better quality of counseling. A 2010 review by WHO and the Global Health Workforce Alliance identified 19 studies which assessed CHW knowledge, attitudes, and practices [20], but only one included data on frequency of CHW recall of critical MNCH content [21]. However, details on the content of knowledge domains assessed are not described, rendering comparison with our study difficult.

In the absence of global standards for CHW knowledge and a wider array of examples in the literature [20], we used the Integrated MNCH CHW Program guidelines and job aids as a reference point and compared MNCH CHW knowledge against health center providers and CHWs interviewed prior to the program’s start in 2011. Among these three different populations, CHWs interviewed in 2011 had a mean overall knowledge score of 64% as compared to 50% for MNCH CHWs and 48% for RCH health center providers. While these single-point estimates do not consider the time lapse between RCH provider training and interview, the finding of lower knowledge scores among RCH health center providers compared to MNCH CHWs is surprising given the longer pre-service training of RCH providers. The performance of CHWs interviewed in 2011—a cadre of providers older in age by a median of 9 years and for whom only 17% had secondary school education or higher—suggests the potential for CHWs existing within the community and/or trained as part of prior vertical programs independently of education levels to be utilized as CHW candidates if they are able to meet certain competency requirements.

Beyond the contextualization of knowledge scores against those observed for other providers, we note that our data do not allow us to link knowledge scores to outcome and impact level indicators. Further, findings from Morogoro Evaluation Project (MEP) facility assessment activities in 2012 suggest that provider knowledge may not translate to improvements in the content of services provided. Rather, they suggest greater complexities may influence service delivery extending above and beyond what one “knows,” including provider and client perceptions, client characteristics, and the availability of provider time for service delivery given the high patient volume and competing time demands, among other factors (Quality of postnatal counseling in primary health care centers in Morogoro, Tanzania: effects of additional training and supervision, submitted for publication 2015) [13]. The poor quality of ANC and PPC in health center services also raises concerns about the implications of CHW efforts to generate increased demand for care seeking in health facilities that are often understaffed, overburdened, and ill-equipped (Content and duration of antenatal counseling and associated factors in selected health centers in Morogoro Region, Tanzania, to be submitted). This highlights the need for CHW programs to consider facility-based improvements parallel to the training, establishment, and ongoing support to community-based cadres. The importance of this has been echoed elsewhere in the literature as part of broader calls to recognize the health systems within which CHW programs are embedded [14,22] and evidence which suggests that relationships between CHWs and providers may strongly effect performance.

Studies elsewhere suggest that CHW performance is higher when the CHW to population coverage ratio is lower [14]. Wide variations in MNCH CHW to population were observed because the program sought to train a fixed number of CHWs per village. In the villages where they were established, MNCH CHWs reported providing services to a mean of 186 households, a figure comparable to the 150 recommended by the Millennium Development Villages [23] and an improvement from the 1 to 3438 households covered by CHWs in the same geographic area in 2011. This population to household coverage ratio roughly corresponds to an estimated 1 CHW per 1000 population. When translated into program activities, CHWs would need to make an estimated minimum of 471 home visits annually or 39 visits monthly above and beyond routine pregnancy and delivery surveillance activities. While steady declines were observed in the mean number of monthly CHW home visits from 21 in May to 15 in September 2013 (median 14 to 12, range of 0–207), results suggest that CHWs may be exceeding visits targets for pregnant women and children 1–59 months but falling short during the postnatal period (0–28 days). While the declines in mean household visits per month could be in part attributed to sharing of workload marked by the successive addition of new CHWs (some of whom went to new villages, others to villages where MNCH CHW had already been deployed), it may too reflect a continuing downward trend in outputs often characteristic of program implementation over time. Moving forward, efforts need to be made to use HMIS and population coverage data to improve its quality and use for CHW performance monitoring, reducing variability in data and service delivery outputs across CHWs and over time. Data on the timing of home visits, and in particular their proximity to the date of delivery for postpartum care, also needs to be measured along with reported uptake of facility-based MNCH services.

At the national level, as dialogue continues on how many CHWs to train per village or population, the implications on individual workload need to be considered given the wide variations in village sizes across Tanzania. While we did not observe a significant association between mean households served and mean monthly home visits reported (Pearson’s correlation coefficient of 0.0128, *P* < 0.8), MNCH CHWs provided services for a mean of 2 days per week—effectively part time. For alternative, more intensive CHW models, setting the number of CHWs to a population ratio versus village number may serve to reduce variability in coverage, content, and quality of care.

Assuming a CHW to population ratio of 1 per 1000, the national scale of this CHW model would require 43 625 CHWs to reach all mainland Tanzanians. The feasibility of identifying and recruiting such a high volume of new providers will need to be determined, particularly if required to have secondary school or higher levels of education. The Integrated MNCH CHW Program’s strategy of training an initial group of CHWs in each facility catchment area and later returning to train more may allow for a stepwise approach to implementation which eases the strain on facility providers and allows communities/MoHSW to identify candidates that meet eligibility criteria over time. This also has implications for CHWs already working in the villages, requiring that they re-adjust their catchment area according to the total number of CHW working in the village.

During the time motion data collection, we sought to better understand the competing demands upon MNCH CHWs’ time, which may in turn have implications for service delivery. While few studies have reported on CHW time spent on service delivery [14], findings from an assessment of community health volunteers in Madagascar suggest a correlation between CHW performance and time spent on the job [24]. In our study, beyond exploring the association between time and home visits, we sought to understand the linkage between financial incentives and total time spent per week on MNCH CHW activities. CHWs were observed to work for a mean of 5 h per week, of which less than 2 h was spent on home visits. When considered in context with the financial incentives received, the overall time spent working on MNCH CHW activities compares favorably to the estimated hourly wage earned through time spent on alternative income-generating activities. This may suggest that CHWs’ programmatic inputs directly correspond to the financial compensation they receive through incentives for initial training and attendance of supervisory meetings. Moving forward, if MNCH CHWs are asked to serve in a full-time capacity, the financial incentive structure would need to be adjusted to ensure comparability with their current earning potential in other sectors.

The availability, frequency, and location of supervision and its linkages with CHW motivation and quality of work have been discussed with limited rigor in the literature [14]. Following the identification, training, and deployment of MNCH CHW activities, the Integrated MNCH CHW Program provided ongoing supportive supervision through support to (a) two facility-based providers per facility, who were encouraged to conduct monthly meetings, and (b) quarterly regional/district MoHSW and Jhpiego supervisory visits. The latter were found to occur with less regularity, in part because Jhpiego/MoHSW may have been engaged in the training of subsequent batches of CHWs and unable to simultaneously initiate supportive supervision of those previously trained. Among facility providers, supervision occurred nearly universally every month and was complemented by visits by facility supervisors to CHWs in the community every other month. CHW attendance of supervisory visits was high, a factor which may be attributed to financial incentives disbursed, the amount of which corresponds to ~50% of the average the MNCH CHW’s household income.

Monthly and quarterly supervisory visits focused largely on HMIS registers. Despite this emphasis, inconsistencies were pervasive in CHW recordkeeping and nearly 25% of CHWs did not maintain MCH registers. This raises concerns about the quality of register data and suggests that a review of register format and content may be warranted to reduce complexity and ease routine documentation of home visits by CHWs. Given the added use of registers to facilitate CHW work flow planning, added attention should be paid to reviewing the accuracy of calculations for home visit scheduling and timely execution of these scheduled visits. To overcome some of these barriers, future CHW programs should consider the use of mobile platforms, which provide frontline health workers with simple tablets or mobile phone devices that facilitate client registration, tracking, and workflow planning and, ultimately, can be linked with reminder and alert systems which send messages to clients and can also offer refresher training information [25,26].

### Limitations

Our analyses suggest that elements of the integrated program supporting MNCH CHWs might be appropriate for delivery at scale; however, the evaluation timing and scope limit the conclusions we are able to draw. Given the early nature of implementation, evaluation activities were limited in focus to output level indicators and thus did not generate an estimate of population-based coverage for MNCH CHW activities including the timing of visits during the pregnancy or postpartum period or referral to health facilities. The delayed initiation of community-based activities—3 years following facility-based training of providers—meant that assessments at the facility level preceded community-level implementation. This time lapse of facility-based capacity building and the initiation of community-based activities may explain differences in the provider knowledge scores observed. The effects of community-based activities on increased demand for services, provider time for supervision, and quality of care were not assessed. The Integrated MNCH CHW Program was implemented at a small scale, focusing on the training and supervision of only four CHWs per facility and a sub-sample of total facilities within each district. This model of partial implementation makes it difficult to draw broader conclusions about program feasibility, acceptability, and effectiveness. Further evidence on the ability of CHWs to provide services of high quality at the community level to target populations at critical time points (i.e. 0-3 days following delivery) and at high coverage is needed before recommendations on the appropriateness of the MNCH CHW program for delivery at scale can be made. A more robust evaluation, which considers elements of the quality of CHW counseling, timing, and coverage of home visits along with changes in facility-based utilization, is recommended to inform decision-making on MNCH CHW implementation moving forward.

## Conclusions

This study profiles MNCH CHWs in Morogoro Region and provides evidence on key operational concerns for CHW programming. Further research is required to understand the balance of CHW performance, CHW to population ratio, and incentives before scale up at the national level. The social profile of CHWs was not significantly associated with CHW knowledge or service delivery suggesting that a broader range of community members could be considered as CHW candidates pending community acceptance and fulfillment of competency requirements. Efforts to increase the service delivery outputs of MNCH CHWs will need to counter incentives CHWs have to devote time to alternative, and often more lucrative, income-generating opportunities. Given prior research demonstrating the existing poor quality of pregnancy and postpartum care at the facility level, efforts to scale up CHW programs need to equally prioritize the strengthening of facility support and service delivery. Despite heavy emphasis on registers during supervision, quality of record keeping was poor and the performance indicators measured were not optimal for tracking the timing of service delivery. CHWs, while highly motivated, also had expectations raised by the program for transportation and other financial incentives, which were not met. These early experiences with MNCH CHW implementation have provided insights into possible areas for program improvement and lessons for scale up.

## Endnotes

^a^To guide counseling during MNCH CHW home visits, CHWs are provided with 26 different job aides. During pregnancy, 17 different job aids are recommended for use during three different home visits (Web figure 3). In the postpartum period, 22 jobs aids are suggested for use at different time points during seven home visits recommended up to 5 months postpartum. Nine job aids are suggested for use during follow-up visits recommended from 5 to 59 months following delivery. While assessment of the quality of counseling was beyond the scope of observations, we observed which job aids were administered during home visits.

^b^If median estimates are used, this corresponds to an estimated 19 pregnancies and 17 newborns annually.

## Additional file

Additional file 1: Figure S1. Overview of Integrated Program MNCH CHW rollout per district. **Figure S2.** Mean composite scores for CHW knowledge and reported service provision on maternal and child health care across the continuum of care and for specific services. **Figure S3.** Observed use of job aids during pregnancy home visits (*n* = 37). **Table S1.** Ordered logistic regression models for composite scores for overall CHW knowledge and specific sub-domains of pregnancy, postpartum, newborn care, and child health controlling for gender, date of training, education, age, and assets. **Table S2.** Ordered logistic regression models for composite scores for family planning, infection/injury prevention, malaria, HIV transmission, and nutrition controlling for gender, date of training, education, age, and assets.

## Abbreviations

ANC: antenatal care
JHU: Johns Hopkins Bloomberg School of Public Health
MNCH: maternal newborn child health
MNCH CHW: maternal newborn and child health community health worker
MoHSW: Ministry of Health and Social Welfare
MUHAS: Muhimbili University of Health and Allied Sciences
PPC: postpartum care
RCH: reproductive child health
