# Supplementary material for: Profile, knowledge, and work patterns of a cadre of maternal, newborn, and child health CHWs focusing on preventive and promotive services in Morogoro Region, Tanzania
Source: Hum Resour Health. 2015 Dec 24;13:98. doi: 10.1186/s12960-015-0086-3 (PMC4690304; doi:10.1186/s12960-015-0086-3)
Supplement: Additional file 1: Figure S1. — Overview of Integrated Program MNCH CHW rollout per district. Figure S2. Mean composite scores for CHW knowledge and reported service provision on maternal and child health care across the continuum of care and for specific services. Figure S3. Observed use of job aids during pregnancy home visits (n = 37). Table S1. Ordered logistic regression models for composite scores for overall CHW knowledge and specific sub-domains of pregnancy, postpartum, newborn care, and child health controlling for gender, date of training, education, age, and assets. Table S2. Ordered logistic regression models for composite scores for family planning, infection/injury prevention, malaria, HIV transmission, and nutrition controlling for gender, date of training, education, age, and assets. [file 12960_2015_86_MOESM1_ESM.docx]

**Supplementary Web Figures**

Web Figure 1. Overview of Integrated Program MNCH CHW rollout per district

CHWs (2-4)

CHWs (2-4)

CHWs (2-4)

CHWs (2-4)

CHWs (2-4)

Villages (2)

Villages (2)

Villages (2)

Villages (2)

Villages (2)

Primary Health Center (2 per district)

Dispensary

Dispensary

Dispensary

Dispensary

Dispensary

Villages (2)

CHWs (2-4)

Supplementary Web Figure 2. Mean composite scores for CHW knowledge and reported service provision on maternal and child health care across the continuum of care and for specific services

**Supplementary Web Figure 3. Observed use of job aids during pregnancy home visits (n=37)**

**Web Table 1. Ordered logistic regression models for composite scores for overall CHW knowledge, and specific sub-domains of pregnancy, postpartum, newborn care and child health controlling for gender, date of training, education, age, and assets**

|  | **Overall knowledge** | | | **Pregnancy** | | | | **Postpartum** | | | **Newborn care** | | | **Child health** | | |
| --- | --- | --- | --- | --- | --- | --- | --- | --- | --- | --- | --- | --- | --- | --- | --- | --- |
|  | Odds ratio | 95% CI | | Odds ratio | | 95% CI | | Odds ratio | 95% CI | | Odds ratio | 95% CI | | Odds ratio | 95% CI | |
| **Gender** |  |  |  |  |  | |  |  |  |  |  |  |  |  |  |  |
| Female | 1.04 | 0.64 | 1.70 | 0.80 | 0.50 | | 1.29 | 1.30 | 0.81 | 2.08 | 1.26 | 0.78 | 2.02 | 1.30 | 0.79 | 2.12 |
| **Date of training** |  |  |  |  |  | |  |  |  |  |  |  |  |  |  |  |
| Dec 2012/Jan 2013 | - | - | - | - | - | | - | - | - | - | - | - | - | - | - | - |
| April / May 2013 | 1.33 | 0.64 | 2.76 | 1.63 | 0.77 | | 3.45 | 0.93 | 0.46 | 1.91 | 1.17 | 0.57 | 2.40 | 1.09 | 0.52 | 2.27 |
| July 2013 | 1.78 | 0.88 | 3.59 | 2.10** | 1.02 | | 4.31 | 1.63 | 0.82 | 3.22 | 1.68 | 0.84 | 3.34 | 1.41 | 0.69 | 2.88 |
| **Education** |  |  |  |  |  | |  |  |  |  |  |  |  |  |  |  |
| Partial/completed primary | - | - | - | - | - | | - | - | - | - | - | - | - | - | - | - |
| Secondary/ higher | 0.85 | 0.44 | 1.64 | 0.75 | 0.40 | | 1.41 | 0.97 | 0.52 | 1.79 | 0.79 | 0.42 | 1.48 | 0.52*** | 0.26 | 1.03 |
| **Age** |  |  |  |  |  | |  |  |  |  |  |  |  |  |  |  |
| < 25 years | - | - | - | - | - | | - | - | - | - | - | - | - | - | - | - |
| 25-35 | 0.83 | 0.43 | 1.61 | 0.84 | 0.45 | | 1.57 | 0.95 | 0.50 | 1.79 | 0.95 | 0.50 | 1.81 | 1.23 | 0.63 | 2.42 |
| >35 | 1.10 | 0.49 | 2.44 | 1.24 | 0.58 | | 2.65 | 1.54 | 0.71 | 3.34 | 1.10 | 0.50 | 2.39 | 1.42 | 0.62 | 3.24 |
| **Asset** |  |  |  |  |  | |  |  |  |  |  |  |  |  |  |  |
| Poorest | - | - | - | - | - | | - | - | - | - | - | - | - | - | - | - |
| Poor | 0.44* | 0.24 | 0.80 | 0.51** | 0.28 | | 0.90 | 0.40* | 0.22 | 0.72 | 0.36* | 0.20 | 0.64 | 0.53** | 0.30 | 0.95 |
| Least poor | 0.44* | 0.24 | 0.82 | 0.50** | 0.28 | | 0.92 | 0.53** | 0.29 | 0.96 | 0.36* | 0.20 | 0.66 | 0.67 | 0.36 | 1.23 |
| * p< 0.01, **p <0.05, *** p<0.10 | |  |  |  | |  |  |  |  |  |  |  |  |  |  |  |

|  | **Family Planning** | | | **Infection/ Injury prevention** | | | **Malaria** | | | **HIV Transmission** | | | **Nutrition** | | |
| --- | --- | --- | --- | --- | --- | --- | --- | --- | --- | --- | --- | --- | --- | --- | --- |
|  | Odds ratio | 95% CI | | Odds ratio | 95% CI | | Odds ratio | 95% CI | | Odds ratio | 95% CI | | Odds ratio | 95% CI | |
| **Gender** |  |  |  |  |  |  |  |  |  |  |  |  |  |  |  |
| Female | 1.07 | 0.67 | 1.71 | 0.84 | 0.52 | 1.34 | 0.68 | 0.42 | 1.10 | 0.86 | 0.54 | 1.39 | 1.09 | 0.68 | 1.75 |
| **Date of training** |  |  |  |  |  |  |  |  |  |  |  |  |  |  |  |
| Dec 2012/Jan 2013 | - | - | - | - | - | - | - | - | - | - | - | - | - | - | - |
| April / May 2013 | 1.25 | 0.61 | 2.56 | 1.90*** | 0.92 | 3.92 | 1.03 | 0.49 | 2.17 | 1.45 | 0.71 | 2.97 | 1.71 | 0.83 | 3.50 |
| July 2013 | 1.87*** | 0.95 | 3.67 | 2.24** | 1.13 | 4.43 | 1.17 | 0.57 | 2.39 | 1.64 | 0.81 | 3.32 | 1.96*** | 0.99 | 3.90 |
| **Education** |  |  |  |  |  |  |  |  |  |  |  |  |  |  |  |
| Partial/completed primary | - | - | - | - | - | - | - | - | - | - | - | - | - | - | - |
| Secondary/ higher | 1.52 | 0.81 | 2.85 | 0.79 | 0.42 | 1.47 | 0.98 | 0.51 | 1.89 | 0.90 | 0.48 | 1.70 | 0.91 | 0.49 | 1.72 |
| **Age** |  |  |  |  |  |  |  |  |  |  |  |  |  |  |  |
| < 25 years | - | - | - | - | - | - | - | - | - | - | - | - | - | - | - |
| 25-35 | 1.33 | 0.70 | 2.55 | 0.93 | 0.48 | 1.79 | 0.53*** | 0.27 | 1.02 | 0.90 | 0.48 | 1.72 | 1.02 | 0.54 | 1.94 |
| >35 | 1.07 | 0.50 | 2.28 | 1.25 | 0.57 | 2.73 | 0.60 | 0.27 | 1.33 | 1.50 | 0.69 | 3.30 | 1.22 | 0.56 | 2.66 |
| **Asset** |  |  |  |  |  |  |  |  |  |  |  |  |  |  |  |
| Poorest | - | - | - | - | - | - | - | - | - | - | - | - | - | - | - |
| Poor | 0.88 | 0.50 | 1.55 | 0.50** | 0.28 | 0.89 | 0.64 | 0.35 | 1.15 | 0.50** | 0.28 | 0.89 | 0.48** | 0.27 | 0.87 |
| Least poor | 1.13 | 0.63 | 2.02 | 0.42* | 0.23 | 0.77 | 0.54** | 0.29 | 0.99 | 0.44* | 0.24 | 0.80 | 0.50** | 0.28 | 0.91 |
| * p< 0.01, **p <0.05, *** p<0.10 | | | |  |  |  |  |  |  |  |  |  |  |  |  |

**Web Table 2.** **Ordered logistic regression models for composite scores for family planning, infection/ injury prevention, malaria, HIV transmission, and nutrition controlling for gender, date of training, education, age, and assets**
